# Glutamine supplementation in the critically ill: friend or foe?

**DOI:** 10.1186/cc13879

**Published:** 2014-05-19

**Authors:** Heleen M Oudemans-van Straaten, Arthur RH van Zanten

**Affiliations:** 1 Department of Intensive Care, VU University Medical Center, De Boelelaan 1117, 1081 HZ Amsterdam, the Netherlands; 2 Department of Intensive Care, Gelderse Vallei Hospital, Willy Brandtlaan 10, 6716 RP Ede, the Netherlands

## Abstract

In the previous issue of *Critical Care*, Mori and colleagues demonstrate that glutamine supplementation in mechanically ventilated patients as part of parenteral nutrition increases plasma glutamine concentration and glutamine utilization, but does not mitigate protein degradation and even increases *de novo* glutamine production. Studies suggest that protein degradation is regulated by the degree of inflammation. Immune cells utilize large amounts of glutamine and derive their glutamine requirements from muscle protein degradation. We hypothesize that the effects of glutamine supplementation depend on the degree of inflammation. Infusing large amounts of exogenous glutamine into patients with inflammatory conditions like sepsis and multiple organ failure may not only enhance immune competence, but may potentially augment the inflammatory response and thereby negatively influence outcome.

## Introduction

Critical illness confers muscle wasting. Skeletal muscle serves as a source of amino acids, especially glutamine, for energy and substrates for immune and other cells. Glutamine is considered conditionally essential and supplementation has been used to maintain muscle and immune function during critical illness. In the previous issue of *Critical Care*, Mori and colleagues present their study evaluating effects of intravenous dipeptide L-alanyl-L-glutamine (0.28 g/kg during 20 hours) supplementation on glutamine metabolism [1]. They hypothesized that exogenous glutamine supplementation would increase plasma glutamine concentrations and thereby attenuate endogenous glutamine production. Contrary to their expectation and, although plasma concentrations increased, glutamine appearance rate increased by 14%. Protein degradation was not attenuated as hypothesized. The increased glutamine appearance was due to increased endogenous glutamine production, while the infused glutamine was 'utilized' and did not inhibit proteolysis.

These results add to the confusion around glutamine supplementation. In addition to the lack of effect of glutamine supplementation on protein degradation in this study, glutamine supplementation even increased mortality in the recent and largest randomized controlled trial in critically ill patients up to now [2]. Of note, the latter used extremely high dosages of glutamine in ICU patients with multiple organ failure, while nutrition was hypocaloric. Does the study of Mori and colleagues provide a clue?

## Immune cells, muscle and glutamine

We revisited older publications [3,4] describing that glutamine is highly utilized by lymphocytes, mainly as a source of nitrogen and carbon precursors for synthesis of macromolecules such as purines 3 and pyrimidines for DNA and RNA, and less so as a fuel through complete oxidation. Moreover, lymphocytes utilize glutamine at rates markedly in excess of what is needed for energy and precursors. The authors postulate a likely purpose of this seemingly futile cycling. High rates of glutaminolysis provide ideal conditions for the provision of intermediates for pathways when required under conditions of increased need, such as rapid immune cell proliferation, independent of the momentary glutamine availability. Glutamine availability for this high rate of glutaminolysis in rapidly dividing immune cells is provided via proteolysis in muscles. Furthermore, if lymphocytes are activated, the rate of glutaminolysis is further increased [5]. *In vitro* studies show that interleukin-2 production by lymphocytes and interleukin-1 production by macrophages depends on glutamine concentration (summarized in [4]). This hypothesis could be extrapolated. Infusing large amounts of exogenous glutamine into patients with inflammatory conditions like sepsis and multiple organ failure may not only enhance immune competence, but may potentially augment the inflammatory response and thereby negatively influence outcome. This ‘inflammation hypothesis’ may explain the seemingly contradictory results of studies on glutamine supplementation: positive results in the pre- and perioperative settings or in ICU patients on prolonged parenteral nutrition [6-11], and neutral [12] or negative results [2] when administered during the acute phase of inflammation or severe multiple organ failure.

## Interpretation

The results of the study by Mori and colleagues support this concept [1]. They demonstrated that protein degradation was independent of glutamine plasma concentrations. Protein degradation is likely controlled by the degree of inflammation. Although the additional exogenous glutamine initially increased plasma concentrations, it was utilized thereafter. No insight is provided as to which cells utilized the additional glutamine, but these could be immune, liver or gut cells. Kao and colleagues [13] investigated glutamine metabolism in sepsis and specifically its conversion to citrulline in the gut. They found that splanchnic glutamine extraction was similar in (fasting) patients with sepsis compared to controls, while leucine extraction was increased. They also found that uptake of glutamine by enterocytes was decreased and the fraction used for conversion to citrulline was diminished. Thus, the lower glutamine concentrations in these septic patients were due to increased clearance by the liver and/or other cells, while intestinal uptake was decreased. These findings confirm previous observations in sepsis showing decreased glutamine utilization by the gut [14]. In contrast, in non-septic patients, glutamine supplementation showed beneficial effects on gut mucosa integrity and barrier function [6,7]. The increased glutamine utilization in the study by Mori and colleagues was likely due to uptake by immune cells. The increased *de novo* synthesis may be attributed to an increase in glutaminolysis by glutamine-activated immune cells, which derive glutamine via *de novo* glutamine production.

## Conclusion

Glutamine supplementation in ventilated patients did not decrease muscle protein breakdown and even increased *de novo* glutamine production in the study by Mori and colleagues. The reason for this may be that protein degradation depends on the degree of inflammation. The rapidly dividing immune cells and the liver are the main consumers of glutamine in sepsis, while glutamine utilization in the gut is inhibited. Immune cells derive their increased glutamine requirements from muscle protein degradation, which seems to be regulated by glutaminolysis in immune cells and thereby by inflammatory stimuli. Glutamine supplementation does not mitigate this process. Supplemented glutamine may even stimulate lymphocyte proliferation and cytokine production and thereby increase immune cell glutaminolysis. Increased *de novo* synthesis of glutamine is essential to support these high rates of glutaminolysis. We hypothesize that, although beneficial effects of glutamine provision have been reported in patients following surgery, trauma, burns or bone marrow transplantation [9-11], glutamine infusion in patients with sepsis or multiple organ failure may further enhance the inflammatory response. Interference of glutamine metabolism in this complex inflammatory process may have unpredictable effects and further studies are warranted to unravel glutamine metabolism in critically ill patients to avoid harm and select patients that may benefit from supplementation.

## Competing interests

HMOvS declares receipt of financial support from FreseniusKabi for glutamine determinations in a previous study. ARHvZ declares receipt of honoraria for advisory board meetings, lectures and travelexpenses from Abbott, Baxter, Danone, FreseniusKabi, Nestle, Novartis and Nutricia.

## Authors' contributions

HMOvS and ARHvVZ were responsible for the intellectual content, the drafting and writing of the article. Both gave final approval of the submitted version and will give approval to any revised version.
